# *Berberis vulgaris* fruit extract mitigated apoptosis in experimentally induced testicular ischemia and reperfusion injury in rats

**DOI:** 10.3389/fphar.2025.1514676

**Published:** 2025-04-01

**Authors:** Serdar Yigit, Sakir Akgun, Isa Yesilyurt, Soner Bitiktas, Ergin Taskin, Ali Alper Kahraman, Muhammed Yayla, Pınar Aksu Kilicle, Seyit Ali Bingol, Bengul Ozdemir, Gül Esma Akdogan, Mehmet Ali Karagöz

**Affiliations:** ^1^ Histology and Embryology Department, Faculty of Medicine, Kafkas University, Kars, Türkiye; ^2^ Kafkas University Department of Medical Biology, Faculty of Medicine, Kars, Türkiye; ^3^ Physıology Department, Kafkas University, Faculty of Medicine, Kars, Türkiye; ^4^ Department of Biochemistry, Faculty of Medicine, Ağrı İbrahim Çeçen University, Ağrı, Türkiye; ^5^ Histology and Embryology Department, Kafkas University, Kars, Türkiye; ^6^ Pharmacology Department, Faculty of Medicine, Selcuk University, Konya, Türkiye; ^7^ Department of Biology, Faculty of Sciences and Arts, Kars, Türkiye; ^8^ Department of Urology, Kafkas University, Kars, Türkiye

**Keywords:** *Berberis vulgaris*, caspase-3, bcl-2, ischemia and reperfusion, testes

## Abstract

**Aim:**

The aim was to investigate the possible protective effect of *Berberis vulgaris* extract, which has anti-inflammatory and antioxidant properties, in the testes in an ischemia–reperfusion (IR) injury model by utilizing molecular, biochemical, histopathological, and immunohistochemical methods.

**Methods:**

A total of 56 male rats were divided in to 7 equal groups. Ischemia was induced by taking the testicles out of the scrotum and rotating them 720° and after 3 h reperfusion was induced. *Berberis vulgaris* extract was administered 3 h before ischemia. The reperfusion groups were treated by oral gavage 1 h before reperfusion. Following the 3-h reperfusion period, tissue and blood samples were collected.

**Results:**

Histopathologically, H&E staining showed disruption in the seminiferous tubule structure in the ischemia and IR groups SOD levels decreased significantly in the IR group compared to the control group, whereas Berberis vulgaris did not change SOD levels following IR at 300 and 600 mg/kg doses. At the dose of 600 mg/kg, *Berberis vulgaris* significantly increased SOD levels compared to the ischemia group CAT activity was significantly higher in the BV300 and BV600 groups compared to the ischemia group. CAT activity was significantly lower in the IR group compared to the ischemia group (p < 0.001). When compared to the control group, the ischemia group had a roughly 3-fold increase in caspase-3 expression. In the IR group, this ratio was dramatically increased—roughly 5-fold. However, the antiapoptotic gene Bcl-2 expression was significantly decreased in both the ischemia and IR groups compared to the control group.

**Conclusion:**

*B. vulgaris* plant extract may have a protective effect against testes IR injury. Further molecular studies are needed to clarify this protective effect.

## 1 Introduction

Testicular torsion is a common medical emergency that can occur in men of all ages, but is especially common in early ages. Early surgery is the most common treatment for testicular torsion. Even if testicular detorsion is performed surgically, testicular damage is observed in 50% of men. Delays in diagnosis and treatment cause testicular loss (Li, 2022; Wei and Huang, 2022). The testicle can heal if detorsion is done within six hours of the commencement of symptoms (Sarac et al., 2019). Following the existing damage caused by ischemia in the testicle, restoration of blood flow after detorsion may cause an increase in tissue damage due to reperfusion. The term “ischemia-reperfusion” (IR) injury refers to this process (Cvetkovic et al., 2015). During the IR process, the amount of reactive oxygen species (ROS) increases excessively in the tissue. Reactive oxygen species such as superoxide anions, nitric oxide, hydrogen peroxide, hypochlorous acid, hydroxyl radicals, and singlet oxygen can damage cellular components such as lipids, proteins, deoxyribonucleic acid, and carbohydrates, causing disruption of cellular viability and even cellular death (Wei et al., 2022). Supportive treatment is used to prevent the negative effects of postsurgical intervention and IR damage. Some protocols used are aimed at reducing oxidative stress. For this purpose, improving antioxidant systems in organ damage caused by IR has effects on reducing IR damage. For this reason, the therapeutic effectiveness of many antioxidant agents has been demonstrated. In particular, plant-derived antioxidant substances are agents used against ROS due to their cost, accessibility, and fewer complications than drugs (Aldemir et al., 2012; Shokoohi et al., 2018). *Berberis vulgaris* is a medicinal plant species in the family Berberidaceae that contains many flavonoids (anthocyanins), phenolic compounds, alkaloids, and organic acids. The most prominent alkaloid in *B. vulgaris* is berberine. Berberine has been reported to have several effects including hypoglycemic, hypotensive, antioxidant, anti-inflammatory, and hypolipidemic. It inhibits the increased production of ROS, which is the leading cause of IR injury; reduces oxidative stress; and decreases the release of apoptosis-inducing factors and the proapoptotic factor cytochrome c in mitochondria that cause apoptosis. Berberine has also been shown in the literature to have antiapoptotic effects against ischemia (Imenshahidi and Hosseinzadeh, 2016; Abushouk et al., 2017; Rahimi-Madiseh et al., 2017). The aim of our study was to examine the protective effects of berberine against testicular IR injury, the changes in the antioxidants superoxide dismutase (SOD) and catalase (CAT) (which have defense functions against oxidative stress), and the location and expression of bcl-2 and caspase 3 to determine the possible effects of the apoptosis process.

## 2 Materials and methods

### 2.1 Animals

The study was approved by Kafkas University Animal Experiments Local Ethics Committee with decision number KA¨U-HADYEK/2019-158. A total of 56 male adolescent (6-week-old) Sprague Dawley rats weighing 180 g were used in this experimental study. The animals were housed in standard living conditions (temperature: 20°C ± 2°C; relative humidity: 50% ± 10%; light/dark cycle: 12 h/12 h).

### 2.2 Plant extraction

The *B. vulgaris* used in the study was collected from Şenkaya district of Erzurum in May 2021. It was brought to the laboratory and dried there, where there was dry airflow and no direct sunlight, in the dark. The body of the dried samples was ground in a grinder. Ethanol was used as the extraction solvent, and 650 mL of it was put into a boiling flask. Ethanol was chosen as the extraction solvent because it is widely used and has strong solvent properties. The Soxhlet method was used because it is widely used and the extract yield is obtained most intensively. The solvent was extracted (10-15 siphons) for approximately 10 h until it became clear. The liquid extracts obtained at the end of 10 h were filtered through blue band filter paper for removal of all particles. The filtered extract sample was evaporated in a rotary evaporator at 35°C-45°C. The plant extract remaining in the balloon was kept in a desiccator for 12 h. The extract, which was completely removed from its solvent, was weighed with an accuracy of 0.1 mg, put into the extract box, and stored at +4°C for use in the study (Wang and Weller 2006; Uluman and Kilicle 2020).

### 2.3 Experiment design

The animals were randomly divided into 7 groups (Table 1).The rats were fasted for 12 h before the surgical intervention. Following anesthesia with intraperitoneal 30 mg/kg thiopental sodium, an incision was made in the scrotum of the rats. The testicles were taken out of the scrotum and rotated 720° and ligated with a 4.0 silk suture to create ischemia. Then they were placed back inside the scrotum and it was closed. Reperfusion was induced after 3 h of ischemia by removing the ligation and restoring rotation. *Berberis vulgaris* extract was administered 3 h before the surgical intervention in the ischemia groups (groups 3 and 4). In the IR groups, it was administered by oral gavage 2 h before the end of reperfusion (groups 5 and 6). After 3 h of reperfusion, the experiment was terminated by euthanasia. Testicular tissue and blood samples were collected.

**TABLE 1:** The descriptions of experimental groups.

Name	Descriptions	Abbreviations	n
Group 1	Control	CNT	8
Group 2	Ischemia: The testes were removed from the scrotum, rotated 720°, and tied with a 4.0 silk suture to create ischemia	I	8
Group 3	Berberis vulgaris 300 mg/kg + Testicular Ischemia: *Berberis vulgaris* 300 mg/kg plant extract was given 3 h before ischemia was induced	I + BV300	8
Group 4	*Berberis vulgaris* 600 mg/kg + Testicular Ischemia: *Berberis vulgaris* 600 mg/kg plant extract was given 3 h before ischemia was induced	I + BV600	8
Group 5	Testicular Ischemia + Reperfusion: Ischemia was created for 3 h. After ischemia, reperfusion was performed for 3 h	IR	8
Group 6	Testicular Ischemia + Reperfusion + *Berberis vulgaris* 300 mg/kg: Testicular ischemia was induced for 3 h. After ischemia, testicular reperfusion was performed for 3 h. *Berberis vulgaris* mg/kg 300 was given 2 h before the end of perfusion	IR + BV300	8
Group 7	Testicular Ischemia (3 h) + Reperfusion (1 h) + *Berberis vulgaris* 600 mg/kg: Testicular ischemia was induced for 3 h. After ischemia, testicular reperfusion was performed for 3 h. *Berberis vulgaris* 600 mg/kg was given 2 h before the end of perfusion	IR + BV600	8

### 2.4 Histological and immunohistochemical examinations

The testicular tissue was fixed in 10% formalin for 48 h (Sahin et al., 2021). Serial sections of 5 µm thickness were obtained from each tissue block with a Leica RM2125RTS microtome. Hematoxylin and eosin (H&E) and immunohistochemical (IHC) staining were performed for histologic examinations. Immunohistochemical Bcl-2 (AF6139 China) and caspase-3 (AF6311 China) protocols specified by the companies were applied. Calculations were performed and the average immunoreactivity intensity was reported. Using light microscopy, semiquantitative scoring was carried out. The immunoreactivity of samples from each animal was scored as follows: none (0), mild (1), moderate (2), severe (3), or very severe (4). Findings were photographed with an Olympus BX43 microscope using the software cellSens.

### 2.5 Biochemical examination

Cold 0.1 M phosphate buffer (pH 7.4) was added to the tissue samples at a ratio of 1/10. Tissue homogenates were centrifuged at 4000 × *g* and +4°C for 20 min. Supernatants were obtained. In the supernatants obtained, commercially available Rat SOD (SunLong, Biotech, CAT: SL1341Ra) ELISA Kit levels and Rat CAT (SunLong, Biotech, CAT: SL1084Ra) ELISA kit activity levels were measured.

#### 2.5.1 Procedure for CAT

well in the Microelisa stripplate empty. 10 μL of

 1. Dilution of Standards
2. As a blank control, leave a well in the Microelisa stripplate empty. 10 μL of sample and 40 μL of sample dilution buffer (dilution factor: 5) are added to sample wells. Samples ought to be placed such they do not come into contact with the well wall. Shake gently while mixing well.
3. The plate is incubated for 30 min at 37°C after the membrane has been closed. Dilution: Use distilled water to dilute the concentrated wash buffer (30 times for 96 T).
4. Washing: Remove the membrane of the sealing plate, aspirate and add washing solution. Allow the washing solution to rest for 30 seconds before discarding it. Five times, the washing process is carried out.
5. All wells, with the exception of the blank control well, receive 50 μL of HRP-Conjugate reagent.
6. Incubation following Step 3’s instructions.
7. Washing as detailed in Step 5.
8. Coloring: Fill each well with 50 μL of each chromogen solution (A and B), gently shake to combine, and then incubate for 15 min at 37°C.
9. Termination: To stop the reaction, 50 μL of the solution is put to each well. The well’s hue ought should shift from blue to yellow.
10. The absorbance O.D. at 450 nm can be measured with a Microtiter Plate Reader.

**Table:** 

36 pg/mL	Standard No.1	300 μL original standard +150 μL standard diluents
24 pg/mL	Standard No.2	300 μL Standard No.1 + 150 μL Standard diluents
12 pg/mL	Standard No.3	150 μL Standard No.2 + 150 μL Standard diluent
6 pg/mL	Standard No.4	150 μL Standard No.3 + 150 μL Standard diluent
3 pg/mL	Standard No.5	150 μL Standard No.4 + 150 μL Standard diluent

### 2.6 Sample collection and RNA isolation

The excised rat testicles were resected and stored at −80°C until RNA isolation. A total RNA kit (E2075, EcoTech Biotechnology) was used for total RNA isolation. Isolation was performed as described in the kit protocol. Samples were stored at −80°C until use.

#### 2.6.1 Real-time PCR (qRT-PCR)

The total RNAs obtained were translated into cDNA using Maxime™ RT PreMix (25,082, iNtRON Biotechnology). The reaction was performed as described in the kit protocol. After cDNA samples were diluted (1:2 ratio), the gene expression levels were detected using the Taqman system (AMPIGENE^®^ qPCR Probe Mix Hi-ROX, ENZ-NUC-106, Enzo Life Science). ABI Step One Plus (Applied Biosystems) was used for the qRT-PCR reactions. The expression levels of caspase-3 (Rn00563902_m1) and Bcl-2 (Rn99999125_m1) genes (4,331,182 TaqMan, Applied Biosystems) were analyzed with the endogenous control gene Beta-Actin (Rn00667869_m1). The differences in gene expression between the groups were presented as fold change (2^−ΔΔCt^ method). Figures from the comparative analysis were plotted using GraphPad Prism™ software (GraphPad Software, San Diego, CA, United States).

## 3 Results

### 3.1 Histopathological findings

**FIGURE 1:**
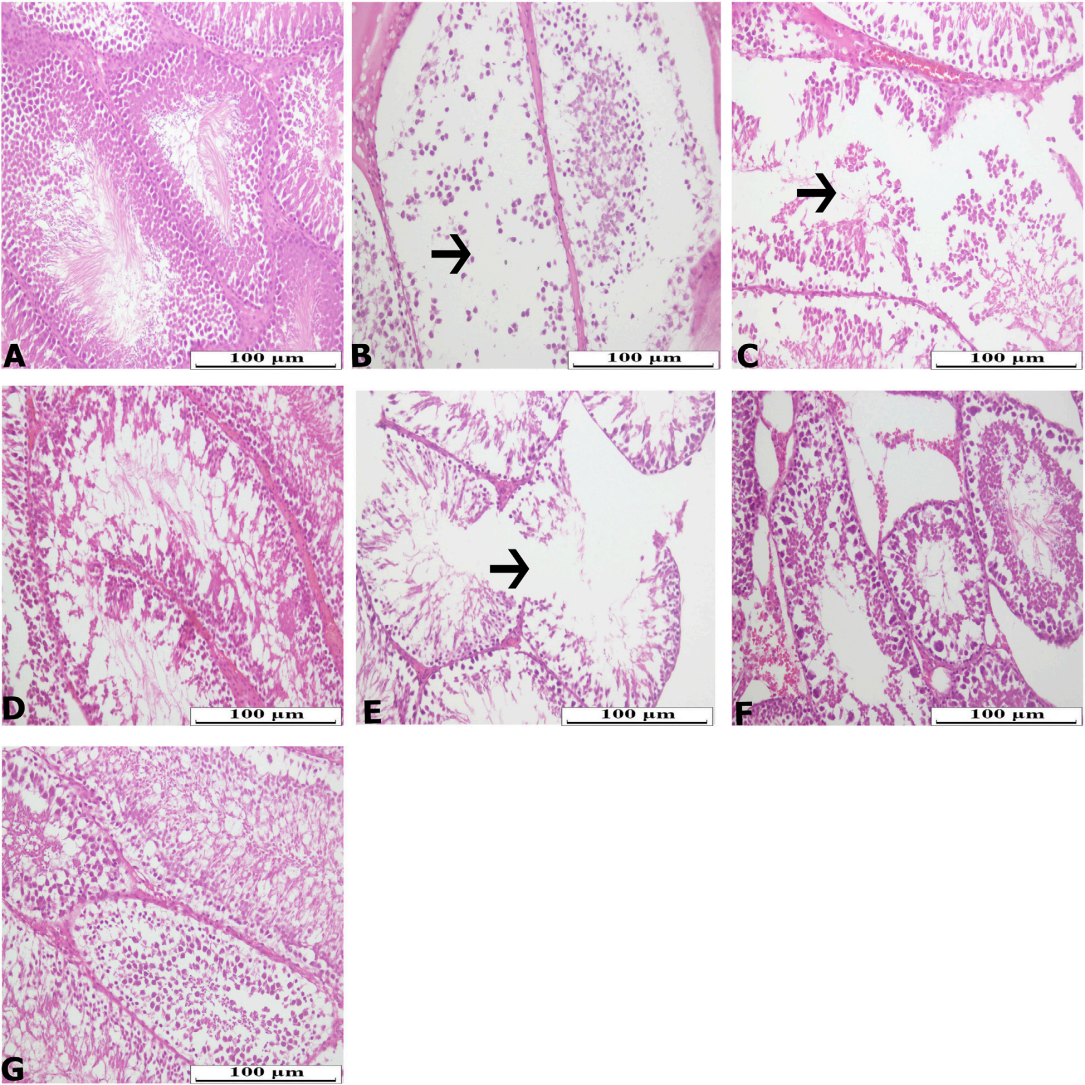
Hematoxylin-Eosin (H&E) staining photomicrographs of all experimental groups. **(A)** CNT, **(B)** I, **(C)** I + BV300, **(D)** I + BV600, **(E)** IR, **(F)** IR + BV300, **(G)** IR + BV600. Arrow indicate seminiferous tubule damage.

H&E staining showed disruption in the seminiferous tubule structure in the ischemia and IR groups (Figure 1). Especially in the ischemia and IR groups, the germinal epithelium was disrupted and the cells forming the spermatogonial series could not be clearly distinguished. It was observed that the germinal epithelium was separated from itself and from the tubular basement membrane. It was observed that spermatogenic cell density decreased and spermatids and spermia decreased in some tubules.

**FIGURE 2:**
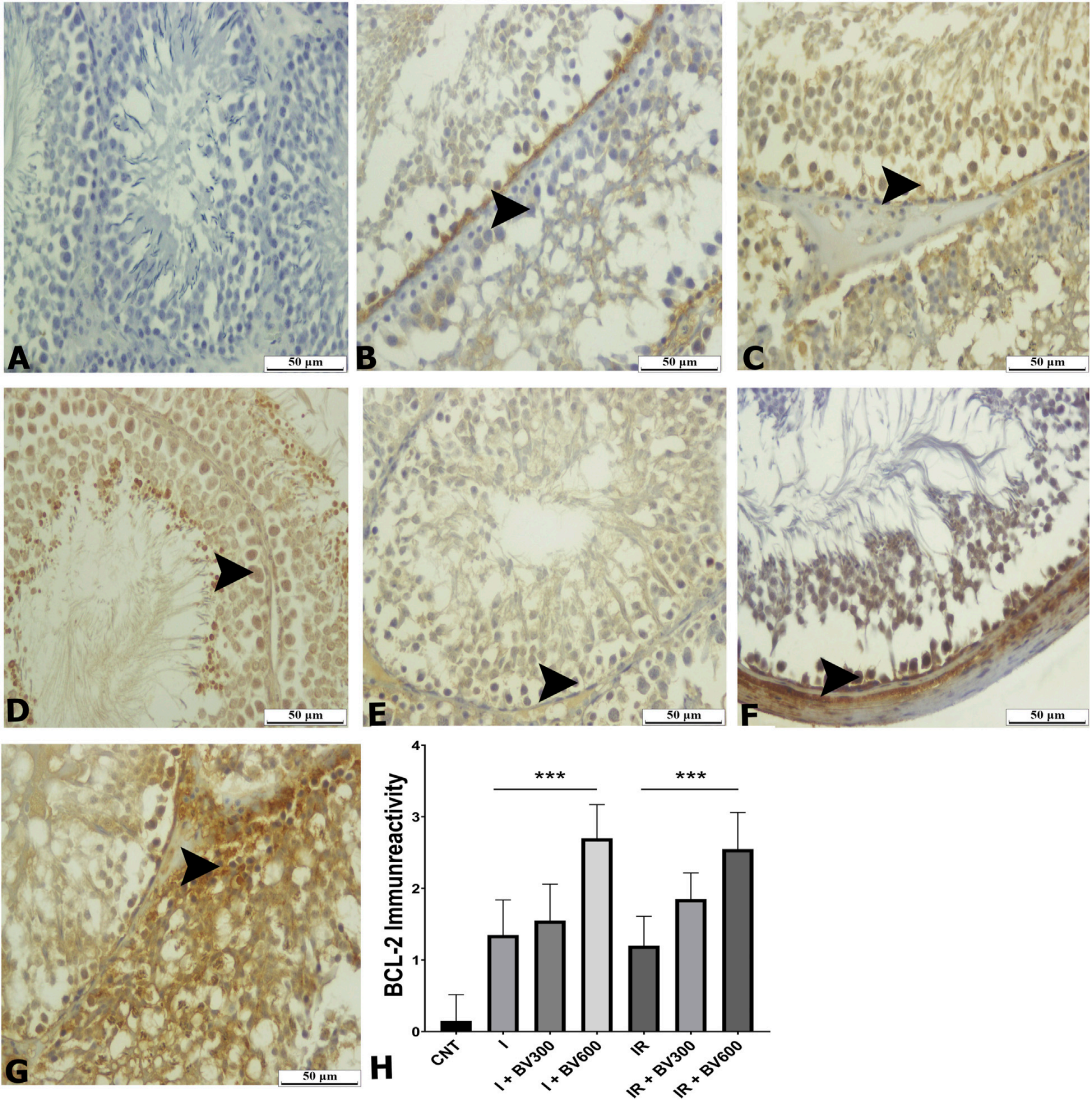
BCL -2 staining photomicrographs of all experimental groups. **(A)** CNT, **(B)** I, **(C)** I + BV300, **(D)** I + BV600, **(E)** IR, **(F)** IR + BV300, **(G)** IR + BV600, **(H)** BCL -2 Immunoreactivity. Results are given as mean ± SD. Kruskal–Wallis test and Dunn’s *post hoc* test were used to analyze data comparing the groups. ***p < 0.001.

Bcl-2 immunoreactivity level was significantly decreased in the ischemia group compared to the control group. *Berberis vulgaris* had no effect at a 300 mg/kg dose, while a 600 mg/kg dose significantly increased immunoreactivity levels compared to the ischemia group (Figure 2). Bcl-2 immunoreactivity levels were significantly decreased in reperfused animals compared to the control group. BV 300 mg/kg and 600 mg/kg doses increased Bcl-2 immunoreactivity levels and reached control group levels.

**FIGURE 3:**
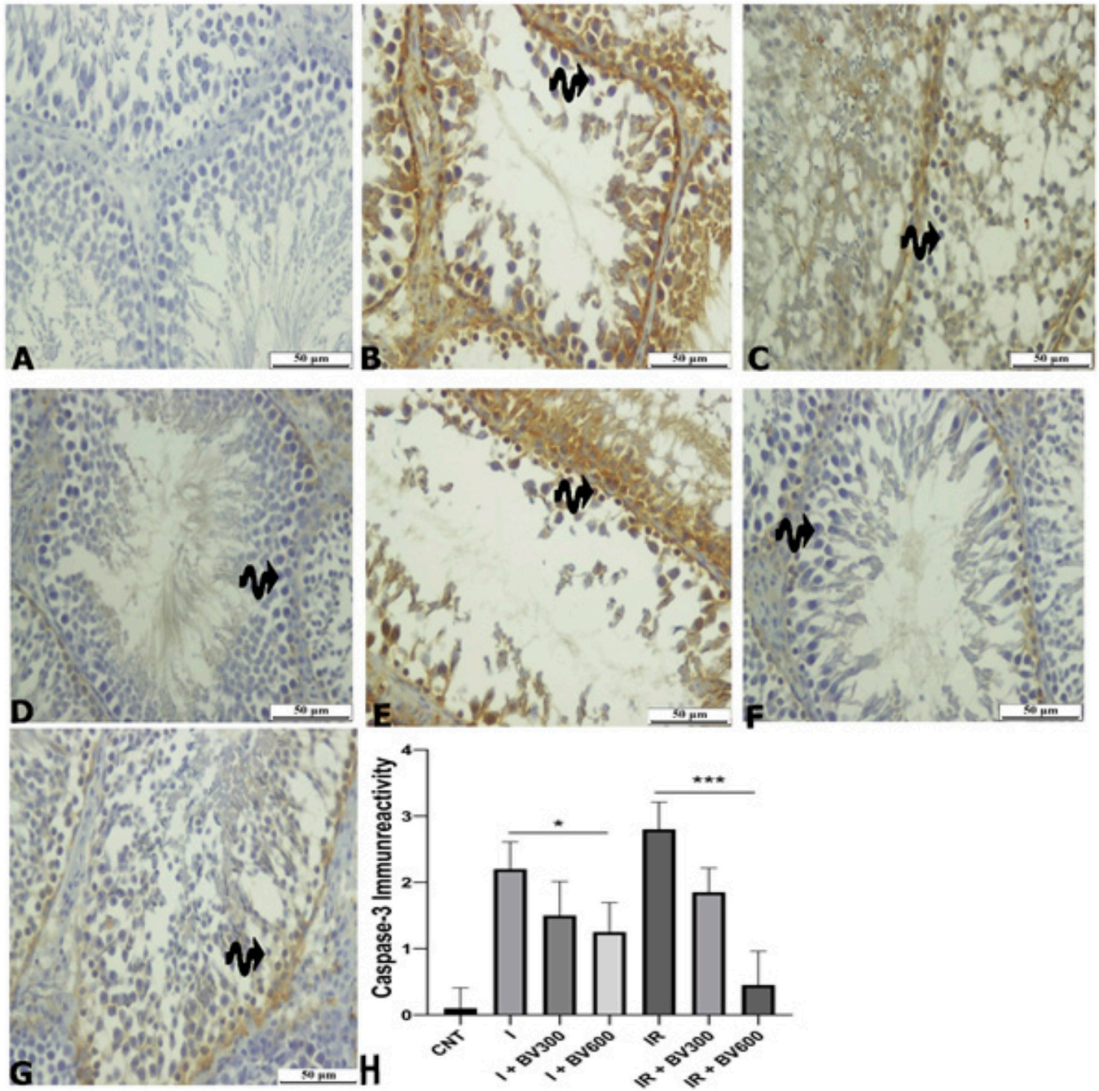
Caspase-3 staining photomicrographs of all experimental groups. **(A)** CNT, **(B)** I, **(C)** I + BV300, **(D)** I + BV600, **(E)** IR, **(F)** IR + BV300, **(G)** IR + BV600, **(H)** Caspase-3 Immunoreactivity. Results are given as mean ± SD. Kruskal–Wallis test and Dunn’s *post hoc* test were used to analyze data comparing the groups. *p < 0.05, ***p < 0.001.

Caspase-3 was substantially higher in the ischemia group compared to the sham group (p < 0.001). BV600 mg/kg dose significantly decreased caspase-3 levels compared to the ischemia group (p = 0.015). While caspase-3 levels were slightly higher in the IR group than in the ischemia group, the difference was not statistically significant. *Berberis vulgaris* 600 mg/kg dose significantly decreased caspase-3 levels compared to the IR group (p < 0.001) (Figure 3).

### 3.2 Biochemical findings

**FIGURE 4:**
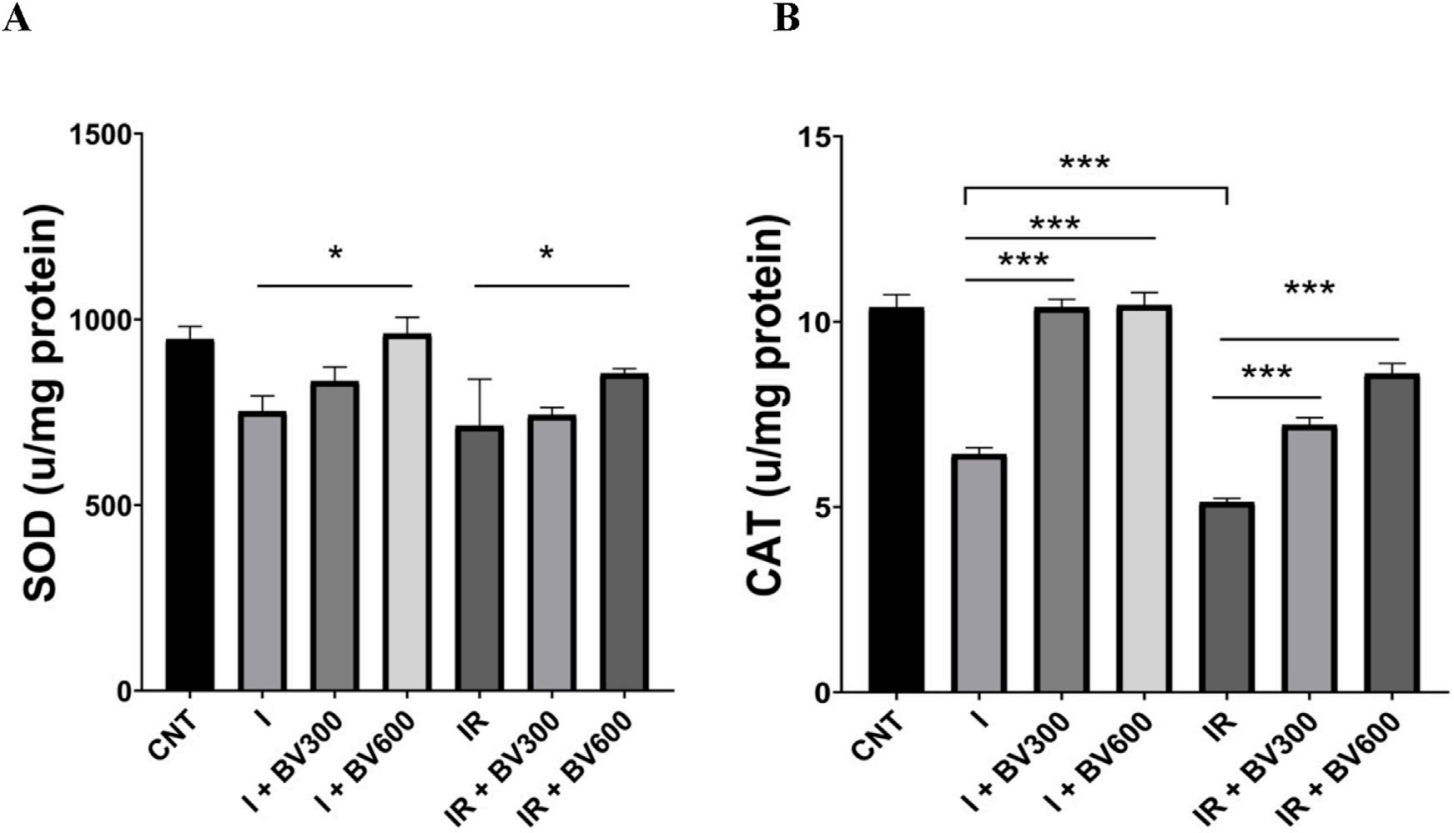
The effect of Berberis vulgaris on SOD **(A)** and CAT **(B)** levels in groups. Results are given as mean ± SD. **(A)** One-way ANOVA and Tukey *post hoc* test were used to analyze data comparing the groups. **(B)** Kruskal–Wallis test and Dunn’s *post hoc* test were used to analyze data comparing the groups. *p < 0.05, ***p < 0.001.

Although SOD levels were decreased in the ischemia group compared to the control group, the difference was not statistically significant (p = 0.70). At the dose of 600 mg/kg, *B. vulgaris* significantly increased SOD levels compared to the ischemia group (p = 0.027), while it had no effect at the dose of 300 mg/kg (p > 0.99). SOD levels decreased significantly in the IR group compared to the control group (p = 0.02), whereas *B. vulgaris* did not change SOD levels following IR at 300 and 600 mg/kg doses (p > 0.99, p = 0.942) (Figure 4).

CAT activity was significantly lower in the ischemia group compared to the control group (p < 0.001). CAT activity was significantly higher in the BV300 and BV600 groups compared to the ischemia group (p > 0.99, p = 0.9997). CAT activity was significantly lower in the IR group than in the ischemia group (p < 0.001). At 300 and 600 mg/kg doses, *B. vulgaris* significantly increased CAT activity compared to the IR group dose dependently (p < 0.001) (Figure 4).

### 3.3 RT-PCR results

**FIGURE 5:**
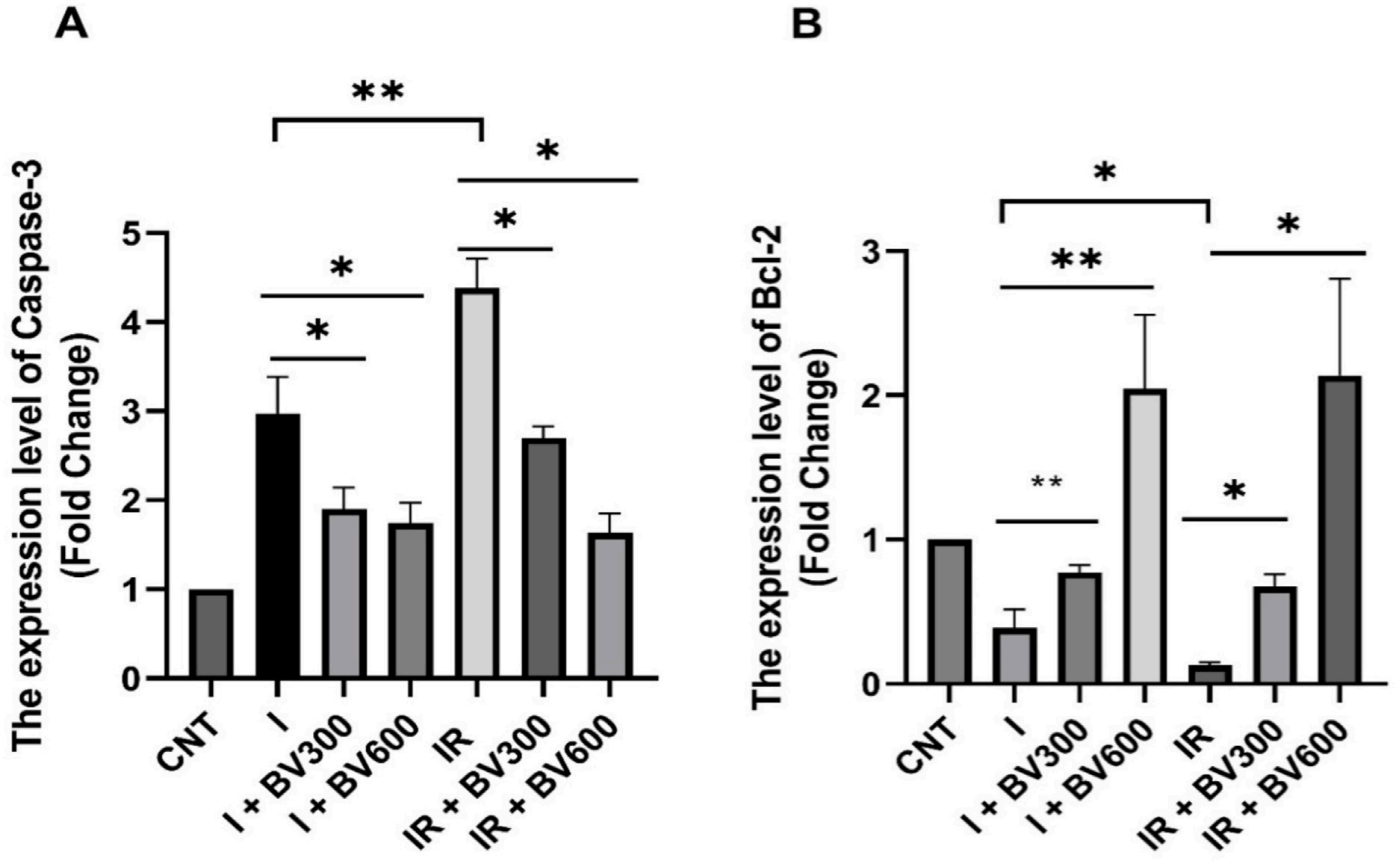
Relative expression levels of caspase-3 **(A)** and Bcl-2 **(B)** mRNAs. Results are given as mean ± SD. Student’s t*-*test was used to analyze data comparing two groups. *p < 0.05, **p < 0.01.

Caspase-3 expression was upregulated about 3-fold in the ischemia group compared to the control group. This ratio increased dramatically by about 5-fold in the IR group. However, the antiapoptotic gene Bcl-2 expression was significantly decreased in both the ischemia and IR groups compared to the control group. In both the ischemia and IR groups, 300 mg and 600 mg of *B. vulgaris* extract significantly downregulated caspase-3 expression levels (Figure 5). The 600 mg application *B. vulgaris* extract was more effective on caspase-3 expression. qPCR analysis revealed a decrease in caspase-3 expression, while Bcl-2 expression increased.

## 4 Discussion

Testicular torsion affects the blood vessels that supply the testicles, reducing blood flow and causing acute ischemic injury. After the detorsion performed to eliminate these consequences, the rapid blood flow caused by reperfusion exposes the testicular tissue to intense oxygen, causing the production of large amounts of free oxygen radicals, which further aggravates tissue damage (Somuncu et al., 2006). Antioxidant treatment before ischemia and after reperfusion is critical to protect testicular tissue from these negative effects and prevent male infertility. Natural antioxidants may be potential protective agents against IR damage. It has been reported that the alkaloid berberine in *B. vulgaris* extract inhibits ROS formation, reduces oxidative stress, and reduces the release of apoptosis-inducing factors. In *B. vulgaris* extract studies, dose of 300 mg/kg and 600 mg/kg was generally used (Taheri et al., 2012; Majeed et al., 2015). With reference to these studies, we adjusted the dose we would give to the animals. It was also reported that berberine has antiapoptotic effects against ischemia by reducing caspase-3 and increasing the Bcl-2/BAX ratio. Berberine has been widely investigated in studies on IR injury because of its antioxidant features. However, there are a very limited number of studies to determine its protective effect against testicular IR injury. In the present study, the aim was to examine the protective effects of *B. vulgaris* against ischemia injury caused by testicular torsion reperfusion injury after detorsion.

Limited number of studies in the literature have reported that berberine has a protective effect by reducing the increased oxidant levels and histological damage caused by testicular IR injury (Gholampour, Malekpour Mansourkhani et al., 2019; Kazaz et al., 2020). In our study, histopathological examinations showed that, in accordance with the literature, the deterioration in the seminiferous tubule structure due to ischemia and IR injury decreased with increasing the dose of *B. vulgaris* extract.

Enzymes like CAT catalyze the synthesis of oxygen and water from hydrogen peroxide, while SOD, the antioxidant system’s first line of defense, catalyzes the formation of oxygen and hydrogen peroxide from a superoxide anion (Hermes-Lima, 2004). In a study investigating the effect of berberine against the damage caused by *Schistosoma mansoni*-induced oxidative stress on testicular tissue, it was reported that berberine showed a protective effect on testicular tissue by decreasing the MDA level and increasing the level of antioxidant enzymes such as SOD and CAT (Dkhil et al., 2014). In addition to this limited number of studies examining the relationship between testicular IR damage and berberine, numerous studies examining the effect of antioxidant systems against IR damage in other tissues such as the heart, liver, kidney and brain reported that it increased the activity of SOD and CAT enzymes (Visnagri et al., 2015; Chu et al., 2017; Jia et al., 2022). In our study, biochemical analysis showed that *B. vulgaris* extract administration significantly increased SOD levels compared to the ischemia group. However, in our study, the administration of *B. vulgaris* extract to rats with IR damage did not change SOD enzyme activity. This may have been due to the increase in the amount of H_2_O_2_ produced by mitochondria due to increased ATP production during adaptation to the stress caused by IR damage. A similar result regarding adaptation to environmental stress was shown by Suzuki et al. (2018). It may also have been caused by the depletion in the antioxidant capacity of SOD due to the depletion in internal energy reserves to cope with stress (Letendre et al., 2012; Sokolova, 2013). A more detailed study is needed to explain this difference. CAT activity, which was decreased considerably after ischemia and IR, were also increased by *B. vulgaris* extract and reached that of the control group, which is consistent with the above-mentioned studies.

Oxidative stress is an important cell death promoter in response to various signals and pathophysiological conditions. ROS accumulation destabilizes the mitochondrial outer membrane and releases proapoptotic factors into the cytosol, inducing caspase activity and cell death (Suen et al., 2008). Cytochrome c binds to apoptotic protease activating factor 1 (Apaf1), facilitating apoptosome formation. Finally, the apoptosome stimulates dimerization of the initiator caspase-9, which cleaves and activates procaspases-3, -6, and -7 (Tait and Green, 2010; McArthur and Kile, 2018). Activated caspase-3 can degrade intracellular structural and functional proteins, resulting in cell death (Yuan et al., 1993). Therefore, Bcl-2 functions as an inhibitor of apoptosis, while caspase-3 is considered an indicator of apoptosis. Although the literature on the antiapoptotic effect of berberine on testicular tissue is very limited, its effects on many other tissues like kidney, heart and brain have been studied in detail. These studies have shown that berberine can inhibit apoptosis by decreasing caspase-3 and BAX expression and increasing Bcl-2 expressi (Wang et al., 2018; Gendy et al., 2023; Binmahfouz et al., 2024). In our study, immunohistochemical analyses showed that Bcl-2 positivity decreased in spermatogenic cells in the ischemia and IR groups in testicular tissue and increased due to the *B. vulgaris* extract dose increase. The Bcl-2 immunoreactivity level was significantly higher and caspase-3 immunoreactivity level was significantly lower in the *B. vulgaris* extract-treated groups compared to the ischemia and IR groups. At the same time, the qPCR analysis demonstrated that Bcl-2 expression was significantly decreased in both the ischemia and IR groups compared to the control group, whereas caspase-3 expression was increased. These effects of ischemia and IR were reversed due to the *B. vulgaris* extract dose increase. The results obtained from the above-mentioned studies are parallel to our results obtained from immunohistochemistry and qPCR results that *B. vulgaris* extract exhibits antiapoptotic effects by reducing caspase-3 levels and increasing Bcl-2 levels.

Our study has limitations. While it provides some valuable information on the protective effect of *B. vulgaris* extract against testicular IR injury, these results are preliminary because *B. vulgaris* plant extract contains chemicals other than berberine. Therefore, more comprehensive studies are needed to reveal the potential of natural compounds to contribute to medical applications. Our results indicating that testicular damage is reduced by *B. vulgaris* extract should be supported by clinical trials.

Considering the results of our study together, it was determined that *B. vulgaris* extract showed a protective effect against testicular IR injury by activating antioxidant and antiapoptotic mechanisms. Since mammalian testes require large amounts of oxygen for continuous active cell division and spermatogenesis, they are susceptible to oxidative stress and oxygen depletion (Davoodi et al., 2020; Balci et al., 2021).

## 5 Conclusion

Immediate surgical treatment of testicular torsion, as well as elimination of testicular IR damage, is critical for preventing male infertility. The improvement of the defect in the seminiferous tubule structure shown by H&E staining, increase in Bcl-2 positivity and decrease in caspase-3 positivity shown by the immunohistochemical evaluation, increase in Bcl-2 expression and decrease in caspase-3 expression in the PCR results, and increase in SOD and CAT enzyme activity shown by the biochemical analysis indicated that the damage and imbalances resulting from ischemia and IR damage were corrected by the administration of *B. vulgaris* extract. The protective effects of *B. vulgaris* extract are promising in this respect.

## Data Availability

The original contributions presented in the study are included in the article/supplementary material, further inquiries can be directed to the corresponding author.
